# Supplementary material for: Identifying the impact of COVID-19 on health systems and lessons for future emergency preparedness: A stakeholder analysis in Kenya
Source: PLOS Glob Public Health. 2022 Dec 21;2(12):e0001348. doi: 10.1371/journal.pgph.0001348 (PMC10021887; doi:10.1371/journal.pgph.0001348)
Supplement: S1 Appendix — (DOCX) [file pgph.0001348.s001.docx]

**S1 Appendix**. Topic guide for key informant interviews

**Identifying the impact of COVID-19 on health systems and lessons for future emergency preparedness: a stakeholder analysis in Kenya**

**Health System Preparedness**

1. How is Kenya’s health system responding to the COVID-19 challenges?
2. How and which health services were the most impacted by the COVID-19 outbreak? Would you describe the impact of COVID-19 on the health system as severe?
3. What is the impact of COVID-19 on these poor vulnerable groups/high-risk populations?  Which are the high-risk population groups most severely impacted?
4. Generally speaking, do you think that the seriousness of this emergency is being underemphasized, treated just right, or exaggerated by the government? Would you consider the government response to the pandemic to be adequate?
5. More broadly speaking about health emergency preparedness and response, what is the general status of national commitment towards improving health security and surveillance (policies/adequate investments/emergency plans)?
6. Can you give specific examples of how the existing health system measures have helped in responding to COVID-19?
7. What were the main shortcomings that were exposed by the emergency? Were they mainly related to information, capacity, supplies, or finances?
8. What lessons did Kenya learn previous health emergencies? Did they help in addressing COVID-19? How could it do better in making sure that it learns from the COVID-19 emergency?
9. Do you feel Kenya’s health system is adequately prepared to address a future health emergency (Why)? Where are the biggest gaps that need to be addressed?
10. Are there any specific countries whose experience in dealing with pandemics you would like to learn more about?

**Financing**

1. Are current resources (finance/health workforce/supplies) adequate to address the emergency?  Are there specific underfunded areas which is affecting the COVID-19 response?
2. What financing measures has the Government taken to ensure essential goods reach the most vulnerable?
3. How has COVID-19 affected current maternal and child health initiatives in the country?
4. Are there steps being taken to ensure continuity of financing of other essential health services? How do you see COVID-19 affecting the health budget, allocations to disease programs and financial planning in the near future?
5. Are domestic investments in pandemic preparedness, surveillance, response and mitigation adequate?
6. From a UHC perspective, do current health financing mechanisms provide any financial protection to Kenyans for services related to COVID-19 and other similar health emergencies? Should this be a priority for the government for future planning?

**Donor Response/Coordination**

1. Who are the main actors that play a key role in managing the emergency (Donors, public health experts, government, NGOs/CSOs) in Kenya?
2. Which international agencies have been the most helpful in responding to this emergency?
3. How would COVID-19 affect donor transitions in Kenya?
4. How has COVID-19 affected the donor funded programs? Was funding from these programs redirected towards COVID-19 response?
5. Given Kenya has highly prevalent diseases like malaria, TB, HIV, how would you expect reallocation of funding towards COVID-19 to take place in a sustainable manner?
6. How are donors coordinating with the government in terms of the COVID-19 response?
7. Do donors plan to adjust transition planning/timelines?  How can they better support emergency preparedness in the future?
