# Supplementary material for: Identifying the impact of COVID-19 on health systems and lessons for future emergency preparedness: A stakeholder analysis in Kenya
Source: PLOS Glob Public Health. 2022 Dec 21;2(12):e0001348. doi: 10.1371/journal.pgph.0001348 (PMC10021887; doi:10.1371/journal.pgph.0001348)
Supplement: Attachment — Submitted filename: Response to Reviewers.docx [file pgph.0001348.s002.docx]

**Author’s Responses to Reviewers**

We thank all the reviewers for assessing our manuscript. We are re-submitting our updated paper for reconsideration. The reviewer’s comments are in black while the author’s responses are in blue.

**Reviewer 1**

Line 104: The author's objective was to understand how the pandemic impacted the health sector and the population more broadly. Consider revising the "impact on population" and focus on the impact on the health sector since population data was not covered in the manuscript.

**Response:** In the results section under the “**Impact of COVID-19 on the health sector and the economy”** on line 311, we included a section describing the impact of COVID-19 on various vulnerable population groups (line 364-line 406). However, we have updated the initial results heading on line 311 to include the population aspect.

Line 109 - Please share some information on how you purposively sampled. Add inclusion criteria for purposively sampling the KIIs.

**Response:** The authors take note of this. We have updated the section to include further information and reference.

Line 121 to 133 - The roles of JO, DO, GK and IB. Add a section for the author's contributions. i.e., GY, KKM, WM roles are not clear.

**Response:** This section has been updated to include the roles of all the authors and provide further clarity (Line 132, 135 and 136). Supplementary information further reflecting authorship contribution using the CRediT taxonomy has been uploaded as a separate file as per the journal’s requirement.

**Reviewer 2**

Overall, the paper addresses a timely topic (that of offering evidence to strengthen health systems and ensure their resilience in future pandemics) while contextualizing this in the Kenyan context. The methods itself appear to meaningfully contribute toward this goal, and writing is relatively clear.

However, the paper largely seems to be lacking in effective use of the literature on this topic. For example, the authors in the abstract suggest links needed with the 6 WHO health system building blocks, but never refer to this framework in the paper or engage with the literature on how these approaches can help to improve pandemic preparedness.

**Response:** Thank you for the comments, the authors take note of them. Regarding the linkage of the paper with the 6 WHO building blocks to present the case for health system preparedness in Kenya, we based our results (both challenges and opportunities) around the framework. Additionally, we developed new themes not covered by the framework but mentioned in the interview to organize our findings. We have also strengthened the literature section to expound on this for more clarity.

Similarly, the paper attempts to draw a link between health systems for health security and UHC but does not draw on recent research on this subject (e.g., Lal et al., Erondu et al., Assefa et al., etc.) -- this is necessary to help further the arguments and structure of the paper. Finally, it may be helpful to define key terms, like UHC (the paper uses this term more as an approach for universal health care, not addressing financial burden as UHC is technically defined) as well as "resilience" of health systems.

**Response:** Thank you for these references which we have now incorporated and expounded in the discussion section.

Minor comments:

- Intro paragraph notes "9 million doses" worldwide -- seems like a typo and should be "billion"? Also, would be good here to note how many went to African continent, since intro makes distinction between global cases and Africa cases of COVID-19.

We have updated this section accordingly with the recent data.

Para 2 -- worldwide is mistakenly repeated after specifically referring to European and American countries ("American" countries is also confusing).

The authors take note of this. We have corrected the section.

- In line 91 -- what are "high" measures?

We defined the stringency of the policy measures taken by governments (whether low, moderate or high), using the metrics defined in the Oxford Coronavirus Government Response Tracker. We have expounded this section in the manuscript for more clarity.

- In the section starting at line 420, title "Opportunities to improve future pandemic preparedness and UHC" -- where does the UHC part come in? This seems to mostly focus on pandemic preparedness. Meanwhile, there is a section soon after on financial protection -- perhaps here would be better to elaborate on UHC.

We have rectified the title and included more information on Universal Health Coverage (UHC).

- In paragraphs starting at line 480, why is infrastructure and human resources considered together, when in previous (1) section, these are separate sub-headings?

We have updated the section as suggested.

- In paragraph starting at line 535, there is a disparity between "majority of the respondents acknowledged that lessons learnt from previous pandemics, including SARS, Ebola, and HIV, helped Kenya leverage the existing systems and policies to fight the COVID-19 pandemic" + "A key lesson learnt for continuity of essential services was to continue having outreaches" vs. your earlier depiction that previous lessons on this were not applied and essential services were severely disrupted during COVID-19 as well -- would be helpful to offer an explanation of why lessons were not learned, otherwise the argument of using lessons learned appears to fall apart.

The argument on the impact of COVID-19 on essential services (Line 318- Line 350) was brought forth to show the initial (almost immediate) reaction by the country to the pandemic. However, this soon changed with key policies being put in place, some of which were informed by previous pandemics. Additionally, various committees were formed and some of the members, including some of those we interviewed for the study were selected based on their initial experience and knowledge on previous pandemics. We also included a section that outlined some of the responses that were brought forth to ensure the continuity of essential services even as more cases were emerging (Line 507- Line 543). However, in the current version of the manuscript, we have updated the section to show that the lessons were incorporated progressively and replaced the quote with one that has more clarity. Thank you for pointing this out.

- The discussion section could benefit from more rigorous analysis and reflection on the findings. As presented, it reads mostly like a summary of the results section, except for a few references linking to similar studies. More meaningful incorporation of other academic research, relevant evidence from other contexts, and implications for what this truly means in terms of specific recommendations would significantly improve the paper.

Thank you for this suggestion. We have now updated the section and included more relevant literature and comparisons from similar studies. We highlight our major findings, their causes and impact and both short- and long-term recommendations that are critical for future pandemic preparedness.
