# Supplementary material for: Identifying the impact of COVID-19 on health systems and lessons for future emergency preparedness: A stakeholder analysis in Kenya
Source: PLOS Glob Public Health. 2022 Dec 21;2(12):e0001348. doi: 10.1371/journal.pgph.0001348 (PMC10021887; doi:10.1371/journal.pgph.0001348)
Supplement: Attachment — Submitted filename: Response to Reviewers.docx [file pgph.0001348.s003.docx]

**Author’s Responses to Reviewers**

We thank all the reviewers for assessing our revised manuscript. We are re-submitting our updated paper for reconsideration. The reviewer’s comments are in black while the author’s responses are in blue.

**Reviewer 1**

Correct the formatting of in-text references 4&5 (Line 59) and references 6&7(Line 61). Both on page 3

**Response:** Thank you for your comment. The authors have taken note of it and updated the section accordingly.

Correct the grammatical error in the sentence that runs between lines 76 to 79. The sentence is " Although initial mitigation measures aimed at limiting the movement of people through 77 lockdowns and quarantines, in a bid to control infections were put in place. These policy 78 directions also affected the access to other health services such as HIV, tuberculosis (TB), and 79 malaria reversing the gains made in curbing these diseases (19)."

**Response:** The authors take note of this. We have corrected the grammatical errors and updated the sentence.

**Reviewer 2**

Updated draft is much stronger (well done!). The discussion still could benefit from a bit more engagement with wider literature, specifically to contextualize the findings with recent advancements/theory in this area. However, the manuscript still strong and offers a unique contribution to the field.

**Response:** Thank you for the comments and suggestion. We have expanded on the literature on the discussion section. Additionally, since our study is majorly focusing on the early responses to the COVID-19 pandemic, we have also highlighted this as a limitation given the evolution of the healthcare space since the COVID-19 pandemic.
